# Deep neural network for prediction of diet quality among doctors and nurses in North China during the COVID-19 pandemic

**DOI:** 10.3389/fpubh.2023.1196090

**Published:** 2023-10-19

**Authors:** Qihe Wang, Haiyun Chu, Huzhong Li, Congyan Li, Shuting Li, Haiqin Fang, Dong Liang, Taotao Deng, Jinliang Li, Aidong Liu

**Affiliations:** ^1^Department of Nutrition Division І, China National Center for Food Safety Risk Assessment, Beijing, China; ^2^Department of Medical Psychology, Public Health Institute of Harbin Medical University, Harbin, China; ^3^Department of Neurology, The First Affiliated Hospital of Jiamusi University, Jiamusi, China; ^4^Health Human Resources Development Center, National Health Commission of the People’s Republic of China, Beijing, China; ^5^Department of General Internal Medicine, Harbin Sixth Hospital, Harbin, China

**Keywords:** COVID-19 pandemic, diet quality, deep neural network, doctors and nurses, China

## Abstract

**Objective:**

The COVID-19 pandemic has placed unprecedented pressure on front-line healthcare workers, leading to poor health status, especially diet quality. This study aimed to develop a diet quality prediction model and determine the predictive effects of personality traits, socioeconomic status, lifestyles, and individual and working conditions on diet quality among doctors and nurses during the COVID-19 pandemic.

**Methods:**

A total of 5,013 doctors and nurses from thirty-nine COVID-19 designated hospitals provided valid responses in north China in 2022. Participants’ data related to social-demographic characteristics, lifestyles, sleep quality, personality traits, burnout, work-related conflicts, and diet quality were collected with questionnaires. Deep Neural Network (DNN) was applied to develop a diet quality prediction model among doctors and nurses during the COVID-19 pandemic.

**Results:**

The mean score of diet quality was 46.14 ± 15.08; specifically, the mean scores for variety, adequacy, moderation, and overall balance were 14.33 ± 3.65, 17.99 ± 5.73, 9.41 ± 7.33, and 4.41 ± 2.98, respectively. The current study developed a DNN model with a 21–30–28-1 network framework for diet quality prediction. The DNN model achieved high prediction efficacy, and values of R^2^, MAE, MSE, and RMSE were 0.928, 0.048, 0.004, and 0.065, respectively. Among doctors and nurses in north China, the top five predictors in the diet quality prediction model were BMI, poor sleep quality, work–family conflict, negative emotional eating, and nutrition knowledge.

**Conclusion:**

During the COVID-19 pandemic, poor diet quality is prevalent among doctors and nurses in north China. Machine learning models can provide an automated identification mechanism for the prediction of diet quality. This study suggests that integrated interventions can be a promising approach to improving diet quality among doctors and nurses, particularly weight management, sleep quality improvement, work-family balance, decreased emotional eating, and increased nutrition knowledge.

## 1. Introduction

Since the beginning of the coronavirus disease 2019 (COVID-19), China policymakers published a series of dramatic containment measures against the COVID-19 pandemic to protect people’s life safety and health. From the strict dynamic zero-COVID policy to the current resumption status, China’s society has gradually returned to normality and resumed work and production. During the COVID-19 pandemic, the medical staff has suffered from a myriad of stressors, such as heavy workload, unstable working setting and hours, strict safety measures, physical exhaustion, and infection risk, which lead to dramatic effects on their work and lifestyles (1). Specifically, poor diet quality not only affects the quality of life, but also leads to adverse health outcomes, such as obesity, metabolic syndrome, diabetes, sarcopenia, and heart diseases (2–10). Consequently, it is necessary to investigate the diet quality of medical staff to provide support to the development of dietary guidelines and nutrition plans for individuals, hospital managers, and national decision-makers.

The Chinese government attaches great importance to people’s health and revised the Dietary Guidelines for Chinese Residents (DGC) in 2022 to guide a healthy diet. Previous studies have pointed out that improving people’s nutrition knowledge could effectively support sound dietary intake and quality among the community or a specific target population (11, 12). Numerous socioeconomic factors including education, income, and occupation influence diet quality. As epidemiologic evidence showed, poor-quality diets were prevalent among individuals from low socioeconomic status (SES) backgrounds (13). Hashimoto et al. found a positive association between education and diet quality and suggested that the diet quality score of high or middle education was higher than low education for both generations (14). Another study showed that diet quality differed by income level, and individuals in the lowest income groups were at greater risk of lower overall diet quality and inadequate nutrient intake (15). A systematic review has provided evidence for the effectiveness of health promotion interventions at the workplace on physical and mental health outcomes (16). The COVID-19 pandemic has brought greater work stress on the medical staff. Work stress and conflict have a great impact on their diet choices and physical health (1). Work–family conflict refers to a lack of overall fit between work and family life, which is a source of stress at the interface of work and family life (17). Participating in multiple roles would inevitably drain substantial time and energy resources, leading to psychological arousal, strain symptoms, emotional eating, and a less healthy diet. As a study showed, work–family conflict and emotional eating were positively related to unhealthy diets (18). A multinational cross-sectional study indicated that job burnout is prevalent among healthcare workers during the COVID-19 pandemic, due to a strong association between SARS-CoV-2 testing, safety attitudes, job role, redeployment, and psychological status (19). The doctors and nurses have to adapt to a new setting during the outbreak, leading to various changes in lifestyles as well as sleep quality (1, 20–24). Smoking, exercise, being overweight or obese, and unhealthy sleep patterns were associated with poor diet quality (25–27). As a type of behavior within a group of various eating behaviors, emotional eating is an important risk factor for poor diet quality and is generally influenced by stress, emotions, lifestyles, and attitudes toward eating (28). Especially in people with obesity, a study has revealed that emotional eaters had lower intakes of vitamins, fiber, folic acid, magnesium, and potassium, while they had a higher intake of sodium, lipids, and fats (29). Additionally, Gacek et al. conducted a study and revealed significant correlations between personality traits and diet quality (30). In practical terms, the pro-healthy diet index showed an increase with increasing extraversion and decreasing neuroticism, while agreeable persons got a lower non-healthy diet index (30). Overall, diet quality is predicated by numerous factors, such as personality traits, socioeconomic status, lifestyles, and individual and working conditions.

Understanding the impact of the COVID-19 pandemic on the diet quality of healthcare workers is crucial. Along with the rapid accumulation of raw information data, machine learning technology plays a key role in modeling algorithms and human life (31, 32). Deep learning technology has demonstrated significant value and outstanding performance in practical applications. Due to their excellent predictability, deep learning models are widely used in various fields, including business, weather, transportation, language recognition, medicine, etc. The nonlinear data transformation performed by hidden layer neurons is the core of deep learning technology. During the training phase, the deep neural network continuously updates the weights of connections between layers, making the results more likely to be close to the target values. Neural networks learn decision functions through a large number of samples. Studies showed that Deep Neural Network (DNN), a powerful tool of machine learning, could provide an automated identification mechanism for various adverse health outcomes, such as body mass index (BMI) changes, diabetic retinopathy, neurological disorders, etc. (1, 33, 34). Given the limited studies focusing on diet and nutrition prediction, applying DNN technology to fill this gap has great benefits. Therefore, this study aimed to develop a diet quality prediction model by applying Deep Neural Network with samples from COVID-19 designated hospitals, and further identify the predictive effects of personality traits, socioeconomic status, lifestyles, and individual and working conditions on diet quality, providing the scientific basis for diet and nutrition improvement among doctors and nurses during the COVID-19 pandemic.

## 2. Methods

### 2.1. Samples

The present study was a cross-sectional study conducted in north China in 2022. Doctors and nurses were randomly recruited from thirty-nine COVID-19 designated hospitals. These hospitals were selected from eight provinces and municipalities in north China, including Heilongjiang, Liaoning, Jilin, Hebei, Shandong, Shanxi, Beijing, and Tianjin. The proportion of participants was defined by the distribution of doctors and nurses from these hospitals. Finally, samples were randomly recruited from these hospitals. Samples eligible for inclusion in this study were Aged ≥18 years old, informed consent, and voluntary participation. Exclusion criteria included voluntary problems, severe diseases, pregnant women, and other special physiological conditions. Eventually, we received 5,013 responses for this study. This study was approved by the Ethics Committee of Harbin Sixth Hospital.

### 2.2. Measures

A dietary assessment survey was conducted with a food frequency questionnaire (FFQ) and then diet quality was assessed with the Diet Quality Index-International (DQI-I). The FFQ was used to measure the food consumption of samples in the last 12 months (35), and the DQI-I focused on four major aspects of a high-quality diet, including variety, adequacy, moderation, and overall balance (36). Variety in the diet was evaluated by overall food group variety (meat/poultry/fish/eggs; dairy/beans; grain; fruit; vegetable) and within-group variety for protein source (meat, poultry, fish, dairy, beans, eggs), with a score of 0–20. Adequacy in the diet was evaluated by vegetable group, fruit group, grain group, fiber, protein, iron, calcium, and vitamin C, with a score of 0–40. Moderation in the diet was evaluated by total fat, saturated fat, cholesterol, sodium, and empty calorie foods, with a score of 0–30. The overall balance in the diet was evaluated by Macronutrient ratio and Fatty acid ratio, with a score of 0–10.

Samples’ socio-demographic information and lifestyles collected in the present study included gender, age, marital status, education, occupation, income, length of employment, overtime, frequency of locked down, vaccination status, smoking, exercise, and BMI. In addition, healthcare worker-patient conflict was assessed by “Have you ever had any conflict with patients during the COVID-19 pandemic?.” Nutrition knowledge was assessed by “Do you have an intimate knowledge of Dietary Guidelines for Chinese Resident?”

Emotional Eating Scale-Revision (EES-R) was used to evaluate negative emotional eating and positive emotional eating, which was developed based on the questionnaires designed by Arnow et al. (37). There were 23 items, and all items were answered on a 5-point Likert scale to measure the level of binge eating. A higher score indicated a stronger desire to eat. The Cronbach’s α coefficient for the scale was 0.964 in the current study.

Pittsburgh Sleep Quality Index (PSQI) (38) was used to assess the sleep quality of doctors and nurses during the COVID-19 pandemic. The PSQI included seven dimensions of sleep quality: subjective sleep quality, sleep latency, sleep duration, habitual sleep efficiency, sleep disturbances, use of sleeping medication, and daytime dysfunction. There were 19 questions in this questionnaire, with a total score ranging from 0 to 21. A higher score indicated poorer sleep quality. In this study, Cronbach’s α coefficient for the questionnaire was 0.917.

Burnout symptoms in this study were assessed by a Chinese version of the Maslach Burnout Inventory-General Survey (MBI-GS) (39). The MBI-GS assessed three dimensions of burnout: emotional exhaustion, depersonalization, and personal accomplishment, with a total of 15 items. Each item was evaluated on a 7-point Likert scale. In the present study, all Cronbach’s α coefficients for the three dimensions were higher than 0.850.

Work–Family Conflict Scale (WFCS) (40) was used to measure the work–family conflict among Chinese doctors and nurses. There were 18 items rated on a 5-point Likert scale. A higher score indicated a higher work–family conflict. The Cronbach’s α coefficient for the scale was 0.913 in this study.

Personality traits were measured using the NEO-Personality Inventory Chinese Version, which was developed by Costa et al. (41). There were five personality dimensions: neuroticism, extraversion, openness, agreeableness, and conscientiousness. A total of 25 items were evaluated on a 5-point Likert scale. Higher scores indicated higher personality trends for the corresponding dimensions. All Cronbach’s α coefficients for the five personality dimensions were higher than 0.850 in this study.

### 2.3. Statistical analysis and machine learning

Descriptive statistics were used to represent the characteristics of doctors and nurses. The associations between diet quality and individual characteristics were tested with the Analysis of Variance (ANOVA) test, t-test, and correlation analysis. All tests were two-tailed, with a statistical significance level set at *p* < 0.05.

This study applied Deep Neural Network (DNN) to develop a diet quality prediction model. R (version 4.2.1) with H2O package was used for performing DNN prediction model. DNN makes a prediction through an input layer, an output layer, and several hidden layers; each layer combines an affine operation and a non-linearity (42). In this study, the output variable was diet quality, and social-demographics characteristics, lifestyles, sleep quality, personality traits, burnout, and work-related conflicts were input variables. Firstly, data were pre-processed with normalization methods. Secondly, we randomly allocated 80% of the sample as the training subset and 20% of the sample as the test subset. In the training phase of the model development, the training subset was used to generate a learned model prediction. In the validation phase, the model was tested with the test subset which would predict the corresponding outcome. Finally, the performance of the diet quality prediction model generated through learning was evaluated by R Squared (R^2^), Mean Absolute Error (MAE), Mean Squared Error (MSE), and Root Mean Squared Error (RMSE).

## 3. Results

### 3.1. Sample characteristics

In this study, a total of 5,013 doctors and nurses were recruited from thirty-nine COVID-19 designated hospitals in north China. Among doctors and nurses during the COVID-19 pandemic, the mean score of diet quality was 46.14 ± 15.08, ranging from 22.00 to 84.00. Specifically, the mean scores for variety, adequacy, moderation, and overall balance were 14.33 ± 3.65, 17.99 ± 5.73, 9.41 ± 7.33, and 4.41 ± 2.98, respectively.

Table 1 shows the samples’ social-demographic characteristics, lifestyles, sleep quality, personality traits, burnout, and work-related conflicts in this study. Of these participants, 2023 (40.4%) were doctors and 2,990 (59.6%) were nurses; 33.2% of participants were males and 66.8% of participants were females. Of these doctors and nurses, 1805 (36.0%) had never been locked down due to COVID-19, and 4,854 (96.8%) participants have already been vaccinated against COVID-19. In underweight, normal, overweight, and obese groups, there were 103 (2.0%), 4,204 (83.9%), 446 (8.9%), and 260 (5.2%) participants, respectively. 2,220 individuals reported having quite good nutrition knowledge. Moreover, the mean scores for negative and positive emotional eating were 37.44 ± 15.04 and 13.83 ± 4.15. The sleep quality score was 7.02 ± 4.17. The mean scores for neuroticism, extraversion, openness, agreeableness, and conscientiousness were 55.61 ± 13.01, 45.85 ± 9.38, 55.40 ± 10.77, 47.83 ± 12.58, and 38.74 ± 11.05, respectively. The work–family conflict score was 50.01 ± 11.30. The mean scores for emotional exhaustion, depersonalization, and personal accomplishment were 15.73 ± 7.39, 9.44 ± 5.85, and 31.77 ± 9.71, respectively.

**Table 1:** Sample characteristics (*N* = 5,013).

Characteristics	Groups	N / M	Percentage / SD
Gender	Male	1,666	33.2%
	Female	3,347	66.8%
Age (years)	<30	730	14.6%
	30–44	2,695	53.7%
	45–59	1,538	30.7%
	≥60	50	1.0%
Marital status	Single	1,090	21.7%
	Married	3,923	78.3%
Education	Junior College & below	240	4.8%
	University	3,078	61.4%
	Postgraduate & above	1,695	33.8%
Occupation	Doctor	2023	40.4%
	Nurse	2,990	59.6%
Length of employment (years)	≤1	189	3.8%
	2–4	428	8.5%
	5–10	1,163	23.2%
	11–15	1,135	22.6%
	16–20	465	9.3%
	>20	1,633	32.6%
Income (Yuan/month)	<3,000	272	5.4%
	3,000–5,000	2,445	48.8%
	5,000–8,000	1823	36.4%
	>8,000	473	9.4%
Overtime (hours/week)	<5	3,611	72.0%
	5–10	989	19.8%
	11–15	175	3.5%
	>15	238	4.7%
Frequency of locked down	0	1805	36.0%
	1	1,342	26.8%
	2	596	11.9%
	3	352	7.0%
	≥4	918	18.3%
COVID-19 vaccines	Vaccinated	4,854	96.8%
	Unvaccinated	159	3.2%
Smoking	No	4,444	88.6%
	Yes	569	11.4%
Exercise	Every day	705	14.1%
	Often(3–5 times / week)	2,738	54.6%
	Occasionally (1–2 times / month)	1,126	22.4%
	Never	444	8.9%
BMI	Underweight	103	2.0%
	Normal	4,204	83.9%
	Overweight	446	8.9%
	Obese	260	5.2%
Nutrition knowledge	No	2,793	55.7%
	Yes	2,220	44.3%
Negative emotional eating		37.44	15.04
Positive emotional eating		13.83	4.15
Poor sleep quality		7.02	4.17
Neuroticism		55.61	13.01
Extraversion		45.85	9.38
Openness		55.40	10.77
Agreeableness		47.83	12.58
Conscientiousness		38.74	11.05
Work–family conflict		50.01	11.30
Healthcare worker-patient conflict	Never occurred	4,000	79.8%
	Occurred	1,013	20.2%
Emotional exhaustion		15.73	7.39
Depersonalization		9.44	5.85
Personal accomplishment		31.77	9.71

As shown in Table 2, the results of ANOVA test, t-test, and correlation analysis indicated that diet quality was significantly associated with gender, age, marital status, education, length of employment, income, overtime, frequency of locked down, exercise, BMI, nutrition knowledge, negative emotional eating, positive emotional eating, sleep quality, neuroticism, extraversion, openness, work–family conflict, healthcare worker-patient conflict, emotional exhaustion, depersonalization (all P<0.05); while no statistical significance of associations was reported on occupation, COVID-19 vaccines, smoking, agreeableness, conscientiousness, and personal accomplishment (P>0.05).

**Table 2:** Associations between social-demographic characteristics, lifestyles, personality traits, work-related conditions, and diet quality among Chinese doctors and nurses (*N* = 5,013).

	F / t/ r	*p*
Gender (male)	−8.015	0.000
Age	8.336	0.000
Marital status	−2.821	0.005
Education	377.430	0.000
Occupation	−0.947	0.344
Length of employment (years)	6.751	0.000
Income (Yuan/month)	18.126	0.000
Overtime (hours/week)	10.635	0.000
Frequency of locked down	15.403	0.000
COVID-19 vaccines	0.344	0.576
Smoking	−0.157	0.875
Exercise	26.230	0.000
BMI	848.765	0.000
Nutrition knowledge	5.356	0.000
Negative emotional eating	−0.153	0.000
Positive emotional eating	−0.126	0.000
Poor sleep quality	−0.736	0.000
Neuroticism	0.034	0.016
Extraversion	−0.055	0.000
Openness	0.068	0.000
Agreeableness	0.024	0.091
Conscientiousness	−0.002	0.876
Work–family conflict	−0.229	0.000
Healthcare worker-patient conflict	−3.026	0.003
Emotional exhaustion	−0.171	0.000
Depersonalization	−0.171	0.000
Personal accomplishment	−0.005	0.701

### 3.2. Diet quality prediction model in Chinese doctors and nurses

**Figure 1:**
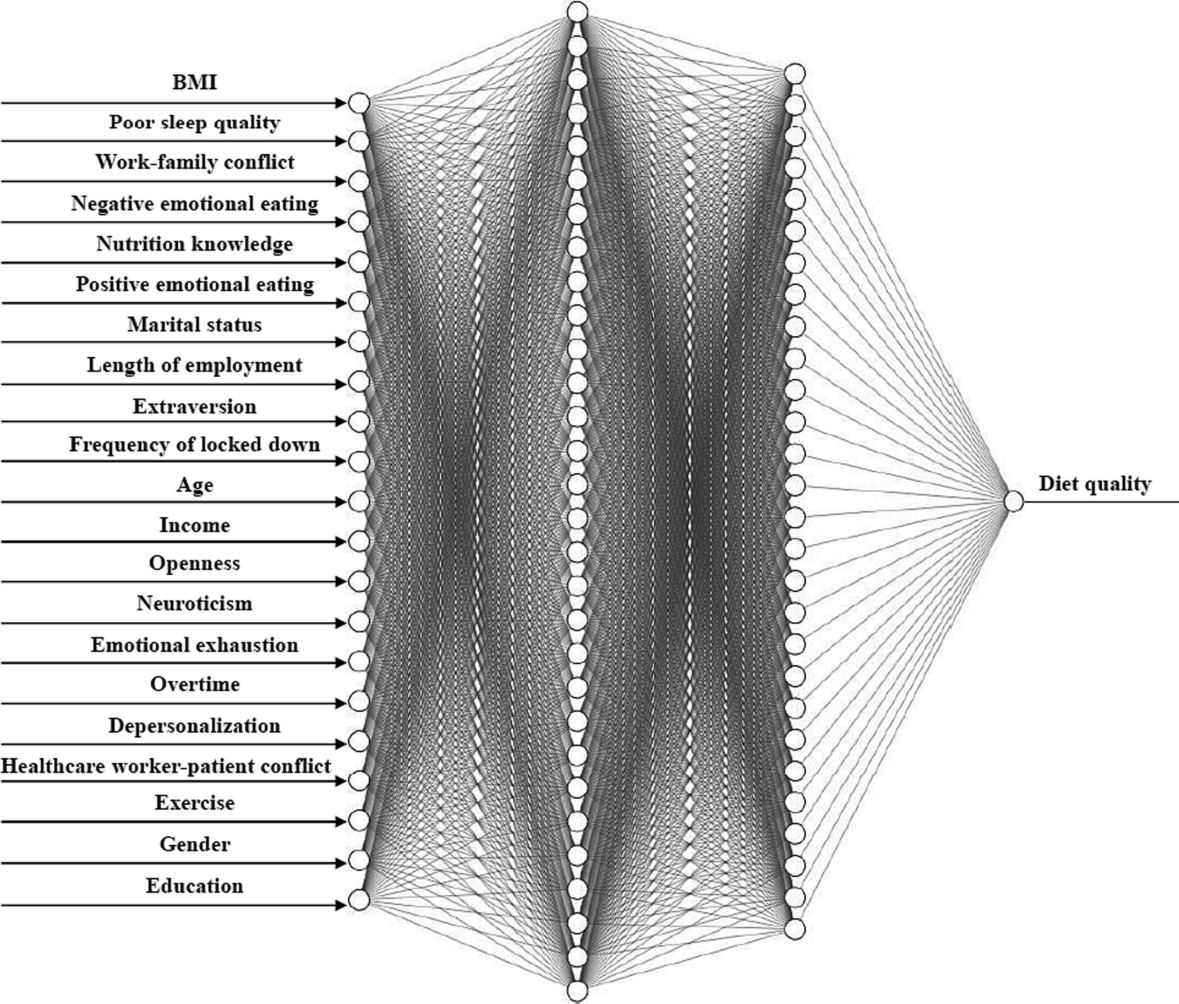
Deep neural network for diet quality prediction.

Based on the results of ANOVA test, t-test, and correlation analysis, this study further developed a Deep Neural Network (DNN) model with a 21–30–28-1 network framework for diet quality prediction, which is presented in Figure 1. For the DNN model, values of R^2^, MAE, MSE, and RMSE were 0.928, 0.048, 0.004, and 0.065, respectively, indicating that the prediction model achieved high efficacy.

**Figure 2:**
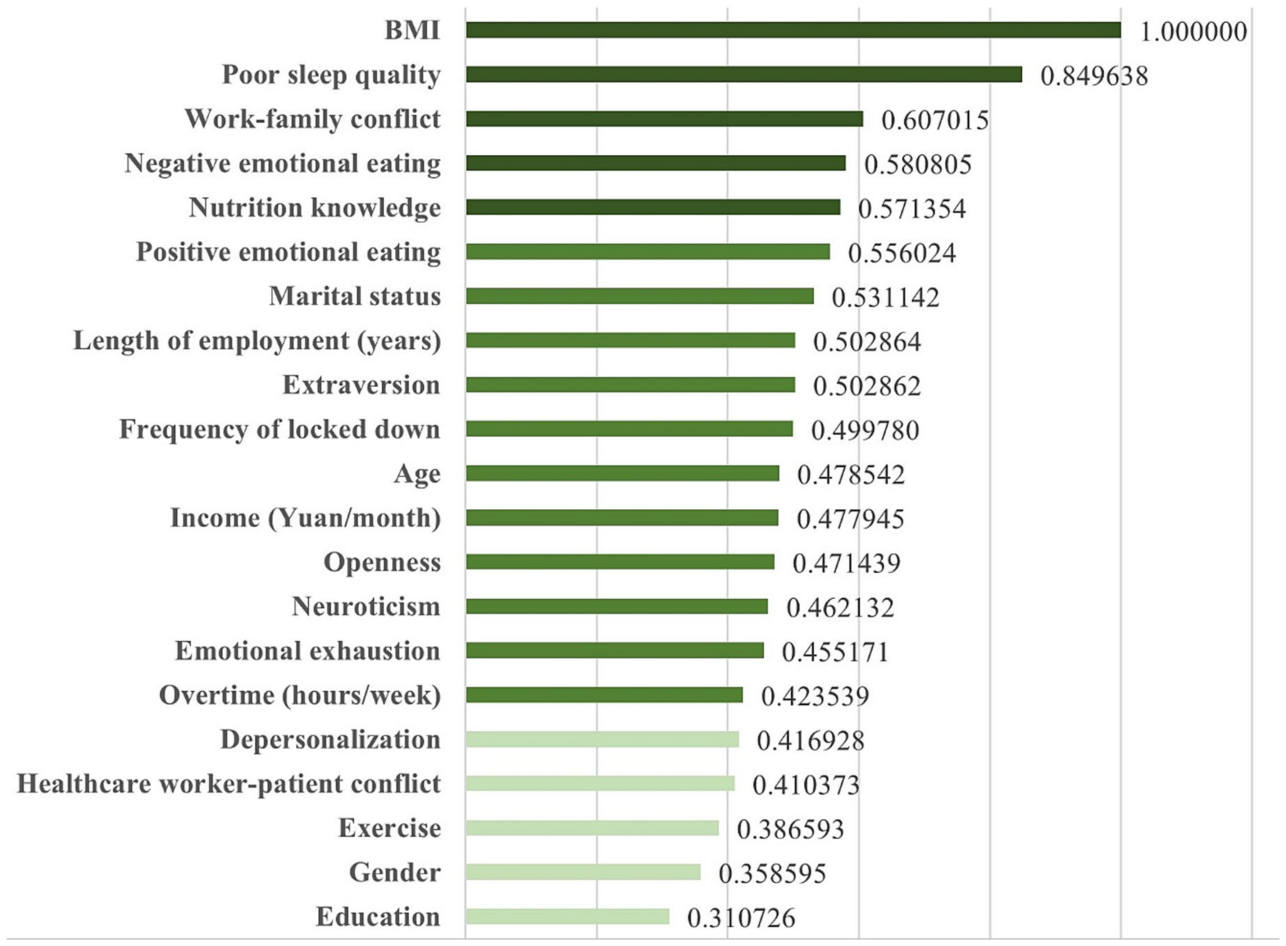
Relative importance of each predictor in the diet quality prediction model.

In addition, as Figure 2 showed, the results of H2O deep learning also revealed the relative importance of each predictor. Among doctors and nurses in north China, the top five predictors in the diet quality prediction model were BMI, poor sleep quality, work–family conflict, negative emotional eating, and nutrition knowledge.

## 4. Discussion

The COVID-19 pandemic has placed unprecedented pressure on front-line healthcare workers, leading to poor health status, especially diet quality. This study recruited 5,013 doctors and nurses from COVID-19 designated hospitals in north China as samples. The mean score of diet quality for all healthcare workers was 46.14 ± 15.08 (the questionnaire score ranging from 0 to 100), indicating poor quality of diets. Specifically, the mean scores for variety, adequacy, moderation, and overall balance were 14.33 ± 3.65, 17.99 ± 5.73, 9.41 ± 7.33, and 4.41 ± 2.98, respectively. Moreover, results showed that diet quality was significantly associated with gender, age, marital status, education, length of employment, income, overtime, frequency of locked down, exercise, BMI, nutrition knowledge, negative emotional eating, positive emotional eating, sleep quality, neuroticism, extraversion, openness, work–family conflict, healthcare worker-patient conflict, emotional exhaustion, depersonalization. Furthermore, the current study developed a Deep Neural Network (DNN) model with a 21–30–28-1 network framework for diet quality prediction, explaining how multiple factors, including work conditions, socioeconomic status, lifestyles, and personality traits, could collectively predict the diet quality of doctors and nurses during the COVID-19 pandemic. In the DNN model, 21 factors were identified as input features, and this deep neural network includes two hidden layers. The DNN model achieved high prediction efficacy, and values of R^2^, MAE, MSE, and RMSE were 0.928, 0.048, 0.004, and 0.065, respectively. Among doctors and nurses in north China, the top five predictors in the diet quality prediction model were BMI, poor sleep quality, work–family conflict, negative emotional eating, and nutrition knowledge.

Results of this study indicated that BMI was the most important predictor of diet quality among doctors and nurses in north China. Of participants in the current study, 83.9% had a normal weight, and 8.9 and 5.2% were overweight and obese, respectively. This study showed that individuals with overweight or obesity had poorer diet quality than individuals with normal weight. Unhealthy eating patterns are one of the important factors for overweight and obesity, and in turn, overweight or obesity impacts diet quality. Previous studies have suggested that the attention bias to food of individuals with overweight or obesity was different from that of individuals with normal weight (43, 44). Food-related attentional bias triggers craving and overeating, especially for individuals with overweight or obesity. People with a higher BMI tend to engage in unhealthy eating habits, such as eating irregularly, a faster eating speed, and a diet high in sugar, etc. (45). It is worth noting that individuals are in a ‘loop’ in the real world, and there is a meaningful self-reinforcing mechanism. Specifically, most obese individuals tend to have a diet with lower quality, which in turn promotes individual weight gain or loss, leading to changes in BMI over time. During the COVID-19 pandemic, healthcare workers were busy on the front line of COVID-19 prevention and treatment, and there was increasing workplace heavy pressure on them. Based on the results of this study, doctors and nurses who had a higher BMI were more likely to eat various high-sugar, high-fat and high-calorie foods to reward themselves, resulting in poor food quality. In addition, due to the irregular working hours, the eating time and eating speed might be inappropriate, which would lead to unconscious overeating. These findings suggest that healthcare workers with a higher BMI should pay more attention to their eating habits and nutritional structure to improve their diet quality.

Among doctors and nurses, poor sleep quality was the second most important factor for diet quality prediction. During the COVID-19 epidemic, a considerable proportion of healthcare workers experience sleep disturbances (46), such as insomnia, short sleep duration, and poor sleep efficiency. The Chinese government established a joint prevention and control COVID-19 team to lead all designated hospitals and healthcare workers’ scientific response to outbreaks; Moreover, doctors and nurses have to adopt 7*24 on-call to keep patients’ life safe and sound. The results of this study determined the important predictive effect of sleep quality on diet quality among doctors and nurses. Indeed, it is well documented that poor sleep quality adversely impacts dietary behaviors (47–49), including higher intakes of energy, fat, and sweets as well as lower intakes of foods shown to have health benefits, including vegetables, fruits, and whole grains. A study has evaluated the cyclical nature of the sleep-diet relationship in adults and revealed various underlying mechanisms of the relationship between sleep and dietary intakes (50). On the one hand, there were increased opportunities to eat and alterations in appetite-regulating hormones (including leptin, ghrelin, adiponectin, glucagon-like peptide 1, and orexin) due to the sleep restriction and added wake time; on the other hand, increased hedonic drive for foods may explain shifts toward poor diet quality following periods of inadequate sleep (50).

Work–family conflict also greatly affected diet quality and ranked third in the DNN model. Increased stress during the pandemic has changed the nature of workplace and family patterns, leading to several work–family conflicts, such as insufficient time to take care of the families and reduced housework completion. An epidemiological study suggested that work–family conflict was implicated in external eating, unhealthy food consumption, poor eating style, and eating quality (18). With the increasingly severe conflict between work and family, unhealthy eating patterns such as irregular meals, fast meals, night meals, and high-energy snacks in healthcare workers are showing a growth tendency. In fact, the ability to balance work and family has a significant impact on healthy eating patterns, family values, work values, and the division of labor in the household, particularly during public health emergencies. Besides, work–family conflict is not only a predictor in explaining unhealthy eating but also a tendency to emotional eating. As the current study indicated, negative emotional eating was the fourth most important predictor of diet quality during the COVID-19 pandemic. Stress and negative emotions may impact appetite, unhealthy eating, and even eating disorders (29, 51, 52). During the COVID-19 pandemic, an Italian survey declared that almost half of the individuals (aged 18–79 years) felt anxious, and consumed comfort food to feel better (53). Individuals with emotional eating tend to increase intakes of energy and macronutrient and have particular food choices, such as fast-food intake, salty snacks, sweet high-fat foods, or energy-dense foods (29). These findings suggest healthcare workers should make efforts to balance work-family, pay attention to their emotional state, reduce emotional eating, and then improve their overall diet quality.

Particularly, nutrition knowledge has a lot of benefits for diet quality, ranking fifth in the DNN prediction model. Nutrition knowledge could play an important role in the nutrition habits and food consumption of an individual, and then change an individual’s diet quality (12). As previous studies showed, an increase in the nutrition knowledge level is related to an increase in the consumption of essential food groups, such as grains, dairy, meats, beans, and so on (54, 55). Especially during the COVID-19 pandemic, nutritional knowledge was significantly associated with the consumption of healthy foods to ensure a robust immune system (56). A healthy eating project in Poland suggested that increasing individuals’ nutrition knowledge was an important target to improve their lifestyles and dietary quality throughout the whole lifespan (57). Results of the current study have identified the predictive effect of nutrition knowledge on diet quality and suggest that interventions of increasing nutrition knowledge can be a promising approach to improving diet quality among doctors and nurses during the COVID-19 pandemic.

This study sheds more light on diet quality prediction in doctors and nurses with a large sample size (*N* = 5,013) and an effective machine learning technology. Based on all the above discussion, these findings indicate that integrated interventions should be taken to improve diet quality among doctors and nurses, particularly weight management, sleep quality improvement, work-family balance, decreased emotional eating, and increased nutrition knowledge. More specifically, establishing a gym and an emotional release room in the hospital can not only facilitate emotional release but also facilitate weight management, which has a significant effect on reducing emotional eating and unhealthy dietary tendencies. Moreover, it is recommended to set up a lounge within different departments for healthcare workers to have lunch breaks and night shifts. To effectively alleviate work–family conflicts, hospital managers can try the following measures: (1) work arrangements are clearer, including fixed working hours, clear work content and processes, clear division of labor, and performance evaluations that pay more attention to work results; (2) cutting between work and personal environments; (3) improving the construction of hospital affiliated kindergartens, primary schools, and other facilities. In addition to providing nutritious working meals, the hospital cafeteria can also conduct more dietary nutrition education, including promotional slogans, posters, brochures, etc., to further improve the nutritional knowledge of healthcare workers.

## 5. Limitations

Some study limitations need to be noticed. Firstly, all participants were recruited from COVID-19 designated hospitals, leading to a potential sampling bias which might reduce the generalization of these results in other medical settings. Secondly, the self-reporting questionnaires were used in the present study and might cause some evaluation errors. Thirdly, since loss of taste and smell are defining symptoms of COVID-19, the lack of data on infection may be a limitation in developing a diet quality prediction model. The data on COVID-19 infection of hospital workers should be collected in future studies. Finally, future studies should collect more information about the national environment and policy to avoid the limited input characteristics, aiming to predict the diet quality of healthcare workers more accurately.

## 6. Conclusion

From the strict dynamic zero-COVID policy to the current resumption status, the COVID-19 pandemic has placed heavy pressure on front-line doctors and nurses in China. The current study has developed a Deep Neural Network (DNN) model for diet quality prediction in doctors and nurses with a large sample size (*N* = 5,013) in north China. Results showed that poor diet quality was prevalent among doctors and nurses and deep neural networks could provide an automated identification mechanism for diet quality prediction. Personality traits, socioeconomic status, lifestyles, and individual and working conditions have contributed to diet quality prediction. Our DNN model revealed that the top five predictors for diet quality were BMI, poor sleep quality, work–family conflict, negative emotional eating, and nutrition knowledge. The current study supports the scientific basis for sustainable diet and nutrition improvement among doctors and nurses. Integrated interventions can be a promising approach to improving diet quality among doctors and nurses, particularly weight management, sleep quality improvement, work-family balance, decreased emotional eating, and increased nutrition knowledge.

## Data availability statement

The raw data supporting the conclusions of this article will be made available by the authors, without undue reservation.

## Ethics statement

The studies involving humans were approved by the Ethics Committee of Harbin Sixth Hospital. The studies were conducted in accordance with the local legislation and institutional requirements. The participants provided their written informed consent to participate in this study.

## Author contributions

AL designed the study. QW and HC drafted the protocol, analyzed the data, and wrote the manuscript. HL, CL, SL, HF, DL, TD, and JL participated in the data collection and analyzed and discussed the results. All authors contributed to the article and approved the submitted version.

## Abbreviations

COVID-19: Coronavirus disease 2019
DNN: Deep Neural Network
R^2^: R Squared
MAE: Mean Absolute Error
MSE: Mean Squared Error
RMSE: Root Mean Squared Error
BMI: Body Mass Index
